# Dr. Holland on the Philosophy of Animated Nature

**Published:** 1849-10-01

**Authors:** 


Art. IV.—*The Philosophy of Animated Nature, or the Laws and Action of the Nervous System.* By G. Calvert Holland, M.D. London: J. Churchill.

Dr. Calvert Holland's work has more than ordinary claims to our attention. Whether the originality of the facts brought forward, the numerous interesting views which they elucidate, or the extensive knowledge exhibited of the labours of former physiologists, as well as of the laws of nature generally, be taken into account, it will be admitted to be a production of considerable value. We have evidence in every page of an active and comprehensive mind. Independently of the principles which it contains, it has literary qualities of a high order. The style is easy, elegant, and perspicuous; and not unfrequently we find in the volume passages of great eloquence and beauty. The mode in which the author has developed his views, is not, indeed, the least merit of the treatise. The work is singularly free from scientific jargon and professional terms, and is, therefore, easy of comprehension to the general reader. Some portions of it, from the novelty of the facts adduced, and the principles which they explain, are as interesting as a romance.

The author exhibits an accurate knowledge of the labours of other physiologists, and has evidently a mind keenly alive to the phenomena of nature generally. The value of the work does not consist simply in the acuteness of its reasoning or the depth of its reflections, *but in the decidedly practical conclusions to which it leads.*

If the views which he endeavours to establish be correct, they will unquestionably be productive of great advantage to the science of medicine. Dr. Holland brings under consideration a nervous principle, not in an hypothetical form. The existence and the laws of its operations are pointed out as clearly and as satisfactorily as Harvey establishes the fact of the circulation of the blood. No one will be inclined to question the value of the discovery of the latter in regard to medical practice, and there would be just as little reason with respect to that of the former. Of the two discoveries, as a previous reviewer has already well observed—"The discovery of the nervous principle, *or rather the tracing of the operations of a power to which the term nervous is applied,* was surrounded by much greater difficulties than that of the motion of the blood. In the latter instance, the structure of the heart, arteries, and veins, when profoundly studied by the philosophical mind, demonstrated that the blood must have the course which the immortal Harvey has indicated.

He had to deal with matter palpable to the senses; and the wonder is, not that he made the discovery, but that it was not made at an earlier period. It is certain, from the important light which had been previously thrown upon the subject, that the circulation of the blood could not possibly have remained long unknown."

The facts which establish the existence and exhibit the operations of a nervous principle have to be sought, not in the structure of the nerves, or indeed in the structure of any part of the animal system, but in the close observation of a multitude of phenomena, the classification of which gives the first glimpse of the laws according to which the nervous principle acts.

The numerous and interesting facts which the author brings forward to prove that such a principle has an existence, and may be accumulated or dispersed in any organ, are, in our opinion, as satisfactory as the evidence which proves the circulation of the blood.

In the short analysis which we propose to give, we shall in the different extracts from the work, allow the author to state his own views; at the same time it must not be expected, in the brief space which we shall occupy, that a correct idea of the contents of upwards of five hundred pages can be conveyed. The work, to be understood, must be read; for though the author considers a variety of subjects connected with the nervous system, yet they are all inseparably connected by the same general facts and reasoning, they all equally illustrate the existence and operations of a nervous principle, consequently they each reciprocally borrow and reflect evidence of their truthfulness.

No medical work has appeared since the days of Harvey, displaying the same novel and comprehensive views; and certainly none having an equal claim to attention, from their exclusive relations to the treatment of diseases. As we have already remarked, if the views be well founded, they will thoroughly change the prevailing doctrines, not only in reference to nervous affections, but to the complicated evils to which flesh is heir.

The first part of the work is occupied in tracing "*the relations between organs and their functions.*" The author enters fully into this subject, and shows that every portion of the animal system is energetic or otherwise in its action, *cæteris paribus,* according to its structural development. He observes—

"The co-existence of thought and feeling with nervous matter, is as indisputable as motion resulting from the contraction of muscular fibres. To admit the dependence in the one instance, and to deny it in the other, is unphilosophical. The connexion in both is established by the same kind of proof, though it may, indeed, be less obvious to the ordinary observer in the operations of mind than in the phenomena of muscular contraction.

"Thought springs, however, from the action of matter as necessarily as motion from muscular contraction, and the power of each is proportionate to the size and condition of its material instruments.

"The doctrine that mental power is proportionate to matter is as universal in its application as the law of gravitation. It excludes exceptions. The vigorous exercise of a faculty implies an ample organ, and the one will never be found separate from the other."

In this part of the work, the author adduces many interesting facts in regard to changes from time and other circumstances, in the structural condition of the organs of the senses, and shows how invariably the senses are modified in their power according to these particular changes. He further proceeds to prove that the activity of these organs, as well as those of the brain generally, *depends on the amount of the nervous fluid which is associated* with them, and he certainly establishes beyond doubt, that this fluid, the same as the blood, is constantly undergoing changes in its amount in all the organs of the body, and that the extent or soundness of their operations is proportionate to the amount of the nervous fluid which they possess. The whole of the work may be said to be occupied in establishing and illustrating this truth. Its application to the explanation of phenomena is shown to be almost inexhaustible. He observes—

"That a function is proportionate to the capacity of its organ may be laid down as a general rule, and all things being equal, admits of no exception. *The power accompanying any given organic capacity is not, however, a* FIXED *quantity, but is constantly exhibiting modifications, not merely in cases of disease, but in the numerous changes of the nervous system in health;* the nature of which modifications is simply a difference in the amount of nervous energy existing. The acuteness or activity of an organ varies with this amount.

"Whatever sense is excited, the nervous principle, in consequence of which it acts, is increased. In vision it flows from within; outwards, not only in the direction of the visal organs, but towards every portion of the human countenance, producing an expression in harmony with the predominant feelings of the mind.

"That phenomena result, which may be explained on this view, will not be doubted; and we shall prove in the following pages that it is in this manner only that numerous facts, in connexion with the nervous system, admit of a satisfactory elucidation. To deny the transmission of a something along the nerves, which is capable of accumulation and dispersion, is to involve the disputant in difficulties and absurdities.

"We do not imagine that the nervous principle is increased by immediate production, when the organ in which it exists is suddenly aroused to powerful action. This principle will flow where it is required, and the knowledge of this circumstance is fraught with the highest interest to the physiologist. Though rich in speculative materials, it abounds far more in truths of a practical nature.

"It is a law of the animal system, that every predominant action draws to itself the vital energies from parts less excited. We observe this in regard to the circulation, and the fact, though less obvious to ordinary observation with respect to the nervous system, may be equally well established by profound investigations. The phenomena which crowd upon the mind in illustration of it, perplex from their variety."

"The flow of the nervous energy from within, outwards, as exemplified in the action of the visual organs, is much more evident in the feline than in the human species. The eyes of the former, when the animal is enraged, glow with fire and actually emit sparks. Does this arise from the transmission of a something from the internal organs, or is it only a change in the constitution of the nervous molecules, neither diminishing nor increasing the principle by which they are animated? The eyes of the cat when intently fixed upon an object in the dark, become large and brilliant in the extreme. The change is not simple concentrated attention, but accumulated nervous energy by which perception is rendered much more acute."

"The hare, when it first catches the distant sound of the dogs, erects its ears and presents a large surface to sonorous impressions. But this is not the whole of the phenomena. A much more sensitive medium is created for the transmission of sound. The same nervous energy which modifies the form of the external organ of hearing, keeps up an excited action between the brain and the complicated apparatus of the sense, and hence the acuteness which results. We cannot, however, on the present occasion, indulge in the amplification of these views."

Almost all physiologists have contended that the nervous fluid has not the slightest analogy to electricity, and in confirmation of the opinion numerous experiments have been performed, apparently establishing the fact. Dr. Calvert Holland points out very clearly the fallacies which exist in all such experiments. Though he does not assert that the two are identical, he shows that no means have been devised which can be regarded as proving a difference in their properties. The following observations on this point are not without interest. He not only shows the source of the fallacies, but suggests an experiment in which they are entirely obviated, thus leaving to future physiologists, the no easy task of discovering a difference in the properties of the two fluids, if such exist.

"We have, in the preceding remarks, taken it for granted, that the experiments, by which Muller and others endeavour to establish the distinction between the nervous and electric fluid, present, in an unobjectionable manner, the phenomena of the latter. We shall, however, show that they have been performed without a due regard to certain important conditions, and consequently have led to false conclusion. We found on operating on the sciatic nerve of a rabbit, that when one pole was placed upon the nerve, and the other on the muscles of the thigh, the contractions were violent; and further, that when the nerve was tied, or divided, leaving a space of the eighth of an inch between the two ends, one pole being applied above the ligature, or the divided portion of the nerve, and the other in its previous situation, the contractions as stated by Muller, were not in any degree lessened. Care was taken, in all these experiments, to insulate the nerve from the surrounding textures by means of glass.

"We conceived, however, that the electric fluid might possibly, through other channels than the trunk of the nerve, find a passage to the muscles, and, therefore, to obviate all sources of fallacy, we separated the spine, at the fourth lumbar vertebra, from the body, and then divided the limb from the pelvis, so that the two portions might be connected at pleasure by the nerve only. When the two ends of the nerve were in the least degree apart from each other, *the electric fluid did not produce any muscular contractions*, but these were observed as soon as the two ends were placed in juxta-position, clearly proving that in the experiments of Müller and others, the electricity had been transmitted not directly through the nerve operated upon, but through other channels to the muscles. The experiment was most striking and satisfactory. When the space between the two ends of the nerves became moist a slightly conducting medium was established. In order to insure success it is necessary that the glass on which the nerve rests should be perfectly dry."

"The numerous phenomena which have been brought under consideration, in reference to the nervous system generally, whether motion, sensibility, or intelligence, or vital action in any of its various conditions, prove that they depend on the same common principle; and that this is indeed the animating power throughout the extended and unbroken chain of breathing flesh, modified only in its manifestations by the capacity and nature of the structure with which it is associated. All that live, move, and have their being, derive that which is designated life, from the union of this principle with organic matter; and when we consider how difficult it is to determine where the animal ends, or the vegetable begins, and reflect on the character of the functions exercised by the latter, it is impossible to resist the conviction, that they are equally the result of the same agent in combination with material elements. In the plant, we observe the ascent and descent of the sap, and the organs of respiration to fit the circulating fluid to the purposes of nutrition, and the variety of its secretions; and also the season in which it displays its maturity and vigour, in the richness of its efflorescence, in which stage of vegetable life the different sexual organs are developed, the presence of the one being essential to the impregnation of the other, and the effect of the mysterious action is the production of that which secures the continuance of the species. And, if we take into account, that electricity abounds in every plant, and is evolved in the ratio of its vital operations, precisely as in the animal; and further, that it pervades all substances, the air we breathe—the water we drink, the food we eat, the ground on which we move, and everything which falls under observation, the conclusion seems almost inevitable that the same principle is common to the whole universe.

"Does such an idea deprive nature of any of its ennobling views? It displays in beautiful simplicity the laws which an all-wise Spirit has impressed on the creations of his hands.

"The contemplation of the infinite phenomena which may be traced to the operation of the same cause,—from the order in which the planets revolve in their undeviating path, to the unfolding of the seed-leaf in search of light, both equally indebted to the same agent for the manifestation of their powers, cannot fail to exalt and refine the conceptions of the mind. The instruments with which nature works are boundless, but that which gives them their vitality or peculiar properties is one, and accompanies matter in all its diversified forms, itself susceptible of no change—save in the laws of its distribution."

By far the most interesting part of the treatise is that in which the author traces "*the influence of the mental faculties on the conditions of the body.*" The whole is not less distinguished by its originality and practical bearings than by the very varied information displayed, and the not less acute observation of the healthy and morbid conditions of the animal system, as well as of human character, pervading its pages, which are nearly half the work. It is not possible, by a few extracts, to convey a correct or general idea of the subjects discussed, or of the mode of their treatment. To be understood, we again repeat, the book must be read. The author traces the operations of the nervous fluid, under a variety of circumstances, and then proceeds to show in what manner the brain, through the medium of the nervous fluid, influences the form and motions of the body, and the expression of the countenance. He refers to the phenomena which Mesmerism discloses, in confirmation of the justness of his view and reasoning. He observes—

"We will briefly advert to the relations between the mind and the body in the several classes of society, and the modifications of these relations and the mode of their production, from alterations in the character of external circumstances.

"In the peasant, especially in purely agricultural districts, where the physical condition of the masses is low, we have the vacant stare, the gaping mouth, the heavy and scarcely rolling eye, and every bodily motion ungainly. These, however, are in harmony with the mental faculties. There are few objects to excite them. Such only are called into play as are necessary for his daily tasks, and the narrow sphere of his social duties. Sleep is his only relaxation from labour.

"It would be against the order and the laws of nature, if these circumstances produced any other results. Quickness of perception, restlessness of thought, and the firm step of independence, are not the growth of such a soil. The animating principle, which, under more favourable conditions, imparts beauty to the human form, grace and dignity to its motions, and intelligence to its every expression, has here a more circumscribed range of action, or, at least, one much less varied. It has to invigorate the muscular powers. These regulate its current, and are the sources of its expenditure. That which is employed, in a different position of life, in developing the mental faculties, in arousing their noblest efforts, and sustaining their loftiest flights, flows here in the direction of those bodily organs on which labour makes the greatest demand. The principle will not maintain two co-existing predominant actions.

"The importance of this view cannot be too strongly impressed upon the understanding. It has simplicity and truth to recommend it. Its application, in the elucidation of phenomena, is boundless, whether they spring out of the healthy or morbid conditions of the cerebral or corporeal organs.

"It must not be imagined that the agent which ministers to the one class of functions, is, in any degree, different in its properties from that which imparts life to the other. It is one and the same, and pervades all nature. The variety of the phenomena to which it gives rise, is according to the diversity of the instruments with which it is associated.

"The awkward movements of the limbs, and the rounded shoulders, partly the effect of labour and of habit, have relations with the brain, which no education or improved social condition can correct, save, perhaps, the rigorous and unrelaxing discipline of the army, and this only in youth.

"The nervous fluid having long flowed in a copious current from the brain to the muscles in active exercise, cannot be turned from its course by new and more varied motives presented to the mind; or by exercising the body in other pursuits. These circumstances may partially modify the conditions, but here ends their influence. We may as well attempt to divert the swelling flood, as withdraw the nervous principle from its long-frequented paths, so changing its distribution, that it shall awaken the higher mental faculties, give life and spirit to the features, and grace and dignity to the human frame.

"The view which explains these relations and the consequent results on the form and motions of the body, is equally applicable to the varying states of the cerebral organs. They have their relations with the nervous fluid, the modifications in the circulation of which account satisfactorily for numerous interesting phenomena arising from their exercise. The same law applies to the diversified conditions of both. The muscles not only acquire strength, but agility of action, by frequent and animated movements; and equally so the organs of the brain, and from the same cause—*the increased amount of the nervous fluid ministering to their operations.*

"The excited and sustained activity of the intellectual faculties, gives occasionally an energy and comprehensiveness of thought, a lucidity of perception, an aptness of illustration, and a copiousness of expression, that appear more like inspiration than the results of mental exertion. What writer has not experienced these luxurious and elevating moments! The happiest conceptions of the poet and of the philosopher, and the exquisite language in which they are clothed, have been the production of that state of the mind in which it pours forth its riches with an unconscious liberality."

"In the middle classes of society, we observe the influence of similar cases, differing only in the degree of intellect which they excite. We have here the first transition from the imperative requirements of labour, to a more active and comprehensive field of exertion. Commerce and manufactures, science, literature, and the arts, appeal to the higher faculties of the understanding, and they impart an energy and enterprise, a vigour and capacity of thought, which cannot co-exist in any other class. The motives to exertion are various. The pure and exalted love of learning for its own sake, but especially for the advantages with which it is pregnant to humanity, are not the least influential of the considerations which stimulate the brain to activity. The necessity of exertion, and the laudable desire to command the comforts and the luxuries of life, arouse the diversified mental powers; and we see their consequences in the busy and enlivening operations of manufactures—in the extension of commerce, in the improved tone, order, and tendencies of society, and in the vast influence which they exert in diffusing the blessings of civilization, wherever man breathes. It is from these classes that the spirit of improvement mainly takes its rise. It communicates an impulse to those above, which renders their progression imperative; and the same is awakened in those beneath them, by presenting for their emulation the fruits of industry, talent, and perseverance. It is their high function to set thought in motion, and their position is peculiarly favourable for giving it the required direction. They are not enfeebled by the indulgences and apathy of the great; nor is their steadiness of purpose, or energy of action, interrupted by the sensual or grovelling ideas of the artisan.

"There may possibly be certain drawbacks to the truthfulness of this picture—shadows which partially obscure the distinctness of its outlines, but the general consequences which flow from the invigorated understanding, are not substantially affected by them.

"We must not expect to find in the majority of these classes, a cultivated taste, a delicate susceptibility of the elegances and nice proprieties of life—an easy and graceful demeanour—the transition which leads to these, springs out of transmitted and not acquired riches. The struggle and the labour by which these are obtained, leave little leisure, and are seldom accompanied with the disposition to study refinements regarded as trifles, but which are fraught with a peculiar charm and interest. They are the last touches which education gives to the feelings. The mental energies are stimulated by more important considerations, and they are kept in vigorous play by the circumstances which call them into existence."

Dr. Calvert Holland is an original thinker. Everything that proceeds from his pen is entitled to our most patient and respectful consideration. He is disposed to think favourably of mesmerism, and the facts he brings forward illustrative of the view he has taken of this *vexata questio,* certainly are startling, and deserve careful investigation. We confess that we have seen enough to convince our own minds that there is *some truth* in Mesmeric phenomena; but, in saying this, we consider it necessary to protest against its betng supposed that we are Mesmerists in the fullest acceptation of the term. Disbelieving and doubting much of the phenomena attributed to this influence, we would respect those who conscientiously pursue these abstruse inquiries. Lord Bacon says of the ancient alchemists, that although they did not succeed in discovering the philosopher's stone, their apparently profitless speculations were productive of much good; they dug up and pulverized the soil, and thus made it more adapted to the purposes of vegetation. May this not be said of Mesmerism?

